# The effect of fresh gas flow rate and type of anesthesia machine on time to reach target sevoflurane concentration

**DOI:** 10.1186/s12871-016-0294-y

**Published:** 2017-01-19

**Authors:** Hye Won Shin, Hae Na Yu, Go Eun Bae, Hyub Huh, Ji Yong Park, Ji Young Kim

**Affiliations:** Department of Anesthesiology and Pain Medicine, Korea University Anam Hospital, College of Medicine, Korea University, Inchon-ro 73, Seongbuk-gu, 02841 Seoul, Republic of Korea

**Keywords:** Inhalation anesthetics, Equipment design, Ventilation

## Abstract

**Background:**

Anesthesia machines have been developed by the application of new technology for rapid and easier control of anesthetic concentration. In this study, we used a test lung to investigate whether the time taken to reach the target sevoflurane concentration varies with the rate of fresh gas flow (FGF) and type of anesthesia machine (AM).

**Methods:**

We measured the times taken to reach the target sevoflurane concentration (2 minimum alveolar concentration = 4%) at variable rates of FGF (0.5, 1, or 3 L/min) and different types of AM (Primus^®^, Perseus^®^, and Zeus^®^ [Zeus^®^-F; Zeus^®^ fresh gas mode, Zeus^®^-A; Zeus^®^ auto-mode]). Concomitant ventilation was supplied using 100% O_2._ The AMs were connected to a test lung. A sevoflurane vaporizer setting of 6% was used in Primus^®^, Perseus^®^, and Zeus^®^-F; a target end-tidal setting of 4% was used in Zeus^®^-A (from a vaporizer setting of 0%). The time taken to reach the target concentration was measured in every group.

**Results:**

When the same AM was used (Primus^®^, Perseus^®^, or Zeus^®^-F), the times to target concentration shortened as the FGF rate increased (*P* < 0.05). Conversely, when the same FGF rate was used, but with different AMs, the time to target concentration was shortest in Perseus^®^, followed by Primus^®^, and finally by Zeus^®^-F (*P* < 0.05). With regards to both modes of Zeus^®^, at FGF rates of 0.5 and 1 L/min, the time to target concentration was shorter in Zeus^®^-A than in Zeus^®^-F; however, the time was longer in Zeus^®^-A than in Zeus^®^-F at FGF rate of 3 L/min (*P* < 0.05).

**Conclusion:**

Shorter times taken to reach the target concentration were associated with high FGF rates, smaller internal volume of the AM, proximity of the fresh gas inlets to patients, absence of a decoupling system, and use of blower-driven ventilators in AM.

## Background

Technological advancements in anesthesia machines (AM) have enabled rapid and easier control of anesthetic concentration and reduced the risks or errors associated with machine operation. However, previous studies have demonstrated that a significant proportion of critical incidents during anesthesia occur due to “the unfamiliarity of the anesthesiologist with medical devices” [1, 2]. Furthermore, Beydon et al. [3] reported that, between 1998 and 2005, there was an increase in the number of errors made while operating medical devices used in anesthesia and intensive care. Therefore, it is essential that anesthesiologists update their knowledge about AM.

The times required to reach the target concentration of inhalational anesthetics varies greatly. To rapidly reach target concentrations of inhalational anesthetics, anesthesiologists generally use either higher fresh gas flow (FGF) or the overpressure technique. However, the time taken to reach the target concentration is also influenced by patient-, inhalational agent-, and equipment-factors. Some factors that slow anesthetic uptake will hasten the rate-of-rise of anesthetic concentration within the lung. Patient- and inhalational agent-factors that decrease anesthetic uptake actually hasten anesthetic induction and recovery; namely low solubility of blood/gas coefficient in inhalational agents or low cardiac output. In addition, the following equipment-factors affect the time taken to reach the target concentration; FGF rate, internal volume of the AM, composition of the AM, target-controlled anesthesia (TCA) systems, etc. [4–8]. Dräger™ has developed several types of AM, all of which are used in clinical practice; however, from a technical point of view, these AMs differ significantly from each other (Table 1).

**Table 1:** The differences in the compositions and configurations of the anesthesia machines; Primus^®^, Perseus^®^, and Zeus^®^ [12, 13, 15]

	Primus^®^	Perseus^®^	Zeus^®^
Mode of anesthesia	Fresh gas mode	Fresh gas mode	Fresh gas mode (Zeus^®^-F) Auto-mode (Zeus^®^-A)
Type of ventilator	Piston	Blower-driven	Blower-driven
Internal volume of anesthesia machine	4.7 L	2.1 L	2.0 L
Position of the fresh gas inlet (the rank for proximity to patient)	Between ventilator and absorber (2nd)	In front of ventilator and absorber (1st)	Next to ventilator and absorber (3rd)
Presence of a decoupling system	Yes	No	No
Type of vaporizer	Bypass	Bypass	DIVA
Operation mode of vaporizer	Out-of-circle	Out-of-circle	Out-of-circle (Zeus^®^-F) In-circle (Zeus^®^-A)
Existence of TCA with a feedback control system	No	No	Yes

*DIVA* direct injection of volatile anesthetics, *TCA* target-controlled anesthesia

The purpose of this study was to compare, using a test lung, the times taken to reach the target sevoflurane concentration of 4% (2 minimum alveolar concentration [2 MAC]) at variable rates of FGF (0.5, 1, or 3 L/min) and different types of AM (the last generation of Dräger™ AMs: Primus^®^, Perseus^®^, and Zeus^®^ [fresh gas mode or auto-mode]) during concomitant ventilation using 100% O_2_.

## Methods

### Preparation of experiments

This prospective study was performed in vitro using a test lung. Experiments were categorized into 10 different groups at various rates of FGF (0.5, 1, or 3 L/min) and different types of AM (Primus^®^, Perseus^®^, or Zeus^®^ [Zeus^®^ -F; Zeus^®^ fresh gas mode, Zeus^®^-A; Zeus^®^ auto-mode]; Dräger Medical GmbH, Lübeck, Germany; Table 1). Before we began our study, we ensured a routine check of the function and calibration of the anesthetic concentration of the Primus^®^, Perseus^®^, Zeus^®^ of the Dräger Company.

The AMs were connected to an anesthesia breathing circuit (Hudson RCI^®^; Teleflex Inc., Morrisville, NC, USA), with an internal volume of 1.2 L, a reservoir bag (RB) of 3 L, and a test lung (Test lung 190^®^; Maquet Critical Care AB, Solna, Sweden). The test lung had a maximum capacity of 1 L, with an internal volume of zero. Initially, free gas was supplied according to the group’s FGF rate for 10 min to maintain a steady FGF rate within the AM. The test lung was then ventilated in the volume-controlled mode at a tidal volume of 600 mL, a respiratory rate of 12 breaths/min, and an inspiratory: expiratory ratio of 1:2.

To measure the sevoflurane concentration, an external gas analyzer (Primus Infinity Vista XL^®^; Dräger Medical GmbH, Germany) was used to obtain gas samples at a rate of 200 mL/min at the Y-piece of breathing circuit; the gas samples were not returned to the system. Before the study, we ensured a routine check of the function and calibration of the anesthetic concentration of the the external gas analyzer of the Dräger Company. The analyzer had an accuracy of 0.15%, a resolution of 0.1%, and a display range of 0–11% for sevoflurane.

### Classification of the comparison groups

We compared the time to target sevoflurane concentration in two sets of experiments, which were based on the vaporizer operation mode in the AM (i.e., out-of-circle or in-circle vaporizer). In the first set of experiments, we compared the AMs with out-of-circle vaporizers (Primus^®^, Perseus^®^, and Zeus^®^-F); in the second set, we compared the different vaporizer operation modes within the multifunctional AM (Zeus^®^-F with an out-of-circle vaporizer and Zeus^®^-A with an in-circle vaporizer).

### Measurement of the time taken to reach target concentration

In Primus^®^, Perseus^®^, and Zeus^®^-F, the target sevoflurane concentration was defined as 4% (approximately 2 MAC); the vaporizer was set to 6% from a previous setting of 0%. In Zeus^®^ -A, the target concentration was defined as 4%; a target setting of 4% was established from a previous vaporizer setting of 0%. The time to target sevoflurane concentration was measured in seconds. Each experimental trial was repeated 5 times at variable rates of FGF (0.5, 1, or 3 L/min) and different types of AMs. We also calculated the Fi/Ft ratio (Fi; the sevoflurane concentration measured at the Y-piece of breathing circuit, Ft; the target sevoflurane concentration) in all groups.

In the present study, the inspiratory and end-tidal concentrations measured by the gas analyzer were equal, because there was no anesthetic uptake, as there is in humans. We measured and recorded the mean sevoflurane concentration waveform on the gas analyzer. After each experimental trial, we renewed the CO_2_ absorber in accordance with the type of AM (CLIC Absorber 800^+^^®^; Dräger Medical GmbH, Germany). To completely wash out the sevoflurane, we ventilated the AM using 100% O_2_ at an FGF rate of 10 L/min for 30 min; the FGF rate was then lowered to 1 L/min for 5 min. If there was a rebound increase in sevoflurane concentration, the AM was ventilated again at an FGF rate of 10 L/min for 10 min [9].

### Statistical analysis

Statistical analysis was performed using SPSS 20 (IBM Corporation, Armonk, NY, USA). The data are presented as median (95% confidence interval). The times to target concentration were compared among Primus^®^, Perseus^®^, and Zeus^®^-F using the non-parametric Kruskal–Wallis test; *post-hoc* multiple comparisons were conducted using the Tukey test. The times to target concentration were compared between Zeus^®^-F and Zeus^®^-A using the non-parametric Wilcoxon signed-rank test. *P*-values < 0.05 were considered statistically significant.

## Results

We performed two kinds of comparisons depending on the vaporizer operation mode used in the AM: (1) a comparison among Primus^®^, Perseus^®^, and Zeus^®^-F; (2) a comparison between Zeus^®^-F and Zeus^®^-A.

**Fig. 1:**
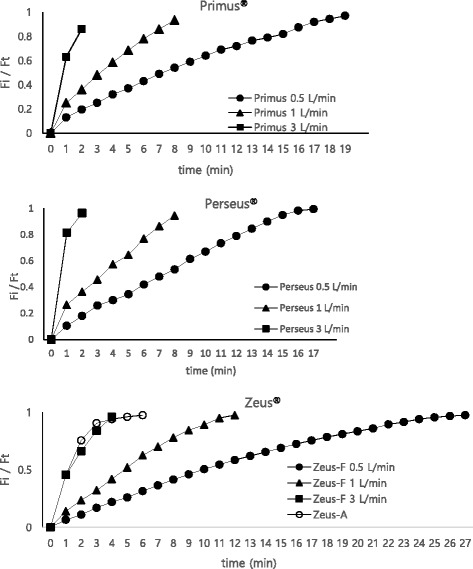
The Fi/Ft ratio curves at variable rate of fresh gas flow (FGF) and different types of anesthesia machine (Primus^®^, Perseus^®^, and Zeus^®^ [Zeus^®^-F; Zeus^®^ fresh gas mode, Zeus^®^-A; Zeus^®^ auto-mode]). The Fi is the sevoflurane concentration measured at the Y-piece of breathing circuit, while the Ft is the target sevoflurane concentration.)

At different FGF rates, but in the same type of AM (Perseus^®^, Primus^®^, and Zeus^®^-F), the time to target concentration shortened as the FGF rate increased (*P* < 0.05; Table 2A, Fig. 1).

**Table 2:** The times taken to reach the target sevoflurane concentrations using a test lung (A) at variable rates of fresh gas flow (FGF) and different types of anesthesia machine (Primus^®^, Perseus^®^, and Zeus^®^-fresh gas mode), (B) at variable rates of FGF and different modes in Zeus^®^ (Zeus^®^-F; Zeus^®^ fresh gas mode, Zeus^®^-A; Zeus^®^ auto-mode) using a lung model. The data are presented as median (95% confidence interval) in seconds. At different FGF rates, but in the same type of AM (Perseus^®^, Primus^®^, and Zeus^®^-F), the time to target concentration shortened as the FGF rate increased (*P* < 0.05; for simplicity, no statistical remarks are included in the table). Identical data are shown for Zeus^®^-F mode in Tables A and B. **P < 0.05 for comparisons with Zeus^®^-F_._ *TCA*; target-controlled anesthesia

A.			
FGF rate	Primus^®^	Perseus^®^	Zeus^®^-F (fresh gas mode)
0.5 L/min	1165 (1150–1185)^†^	920* (883–960)	1590*^†^ (1528–1639)
1 L/min	534 (505–538)^†^	445* (428–474)	705*^†^ (690–729)
3 L/min	155 (149–164)^†^	134* (121–137)	255*^†^ (247–260)
B.			
FGF rate	Zeus ^®^-F (fresh gas mode)	FGF rate	Zeus^®^-A (auto-mode)
0.5 L/min	1590 (1528–1639)	Auto-control of FGF by TCA	380 ** (374–389)
1 L/min	705 (690–729)		
3 L/min	255 (247–260)		

*P < 0.05 for comparisons with Primus^®^_._ † *P* < 0.05 for comparisons with Perseus^®^

**P < 0.05 for comparisons with Zeus^®^-F

However, at the same FGF rate in different types of AM (Perseus^®^, Primus^®^, and Zeus^®^-F), the time to target concentration was shortest in Perseus^®^, followed by Primus^®^, and finally by Zeus^®^-F (*P* < 0.05; Table 2A).

Comparisons between the two modes of the Zeus^®^ showed that the time to target concentration was shorter in Zeus^®^-A than in Zeus^®^-F at FGF rates of 0.5 and 1 L/min. However, at an FGF rate of 3 L/min, the time was longer in Zeus^®^-A than in Zeus^®^-F (*P* < 0.05; Table 2B, Fig. 1).

The Fi/Ft ratio curves from all groups are shown in Fig. 1.

## Discussion

The present study showed that the time to target sevoflurane concentration is shorter at higher FGF rates, and that it varies in different types of AM used (Perseus^®^, Primus^®^, and Zeus^®^-F). In the comparison between Zeus^®^-A and Zeus^®^-F, the time to target concentration varied at variable rate of FGF.

Because of our study design, patient-factor and inhalational agent- factor were excluded from the time to target concentration; that is, we used a test lung and a single inhalational anesthetic (sevoflurane). In this way, this experiment focused on equipment-factor. Kern et al. [7] reported that, in toddlers and newborns, the time to target concentration shortens at high FGF rates, at small internal volumes of the AM, and at high minute ventilations. In the present study, the Primus^®^ [7, 12], Perseus^®^ [13], and Zeus^®^ (Zeus^®^-F and Zeus^®^-A) [5, 6, 14, 15] differed in the following ways: (1) type of ventilator, (2) internal volume, (3) proximity of the fresh gas inlet to the patient, (4) presence of the decoupling system, (5) type of vaporizer, and (6) involvement of TCA (Table 1).

In the present study, given the same FGF rate, the times to target sevoflurane concentration shortened in the order of Perseus^®^, Primus^®^, Zeus^®^-F. Theoretically, the time constant (τ = circuit’s internal volume/FGF rate) characterizes the response time in which a circular breathing system reaches its target concentration [10, 11]. We calculated the circuit volume of each AM (the internal volume of AM + the volume of breathing circuit 1.2 L) and the time constant (the circuit volume/FGF rate) at variable rate of FGF and for different types of AM (Table 3). As the FGF rate increased, the difference among the AMs in terms of the time to target sevoflurane concentration tended to decrease. Importantly, the Perseus^®^ and Zeus^®^ had similar circuit volumes (3.3 L and 3.2 L, respectively) and time constants (6.6 and 6.4 min, respectively, at an FGF 0.5 L/min) (Table 3). In contrast, the Primus^®^ had a circuit volume of 5.9 L and a time constant of 11.8 min at an FGF 0.5 L/min (Table 3). Despite this, there were significant differences in the time to target sevoflurane concentration between Perseus^®^ and Zeus^®^-F (Table 2). We posit that these differences were due to the equipment-factors other than known factors such as the circuit volume and FGF rate [7].

**Table 3:** The circuit volume of anesthesia machine (AM) and time constant at variable rates of fresh gas flow (FGF) and different types of AM

		Primus^®^	Perseus^®^	Zeus^®^-F (fresh gas mode)
The circuit volume of AM (the internal volume of AM + the volume of breathing circuit 1.2 L)		5.9 L	3.3 L	3.2 L
Time constant (the circuit volume/FGF rate)				
FGF rate	0.5 L/min	11.8 min	6.6 min	6.4 min
	1 L/min	5.9 min	3.3 min	3.2 min
	3 L/min	1.9 min	1.1 min	1.0 min

The shortest time to target sevoflurane concentration, obtained using the Perseus^®^, was likely due to the small internal volume of the AM, the proximity of the fresh gas inlet to the patient, the absence of a decoupling system, and the rapid ventilation by a blower-driven ventilator (Table 1). The internal volumes of the AMs, including the CO_2_ absorbers, but without the breathing circuits, are 4.7 L for Primus^®^, 2.1 L for Perseus^®^, and 2.0 L for Zeus^®^ (Zeus^®^-F and Zeus^®^-A; Table 1) [12–14]. In the Perseus^®^, the fresh gas inlet is closest to the patient, followed by Primus^®^, and finally by Zeus^®^ (Zeus^®^-F and Zeus^®^-A; Table 1) [12–14]. Fukuda et al. [16] reported that the proximity of the fresh gas inlet to the patient improves the inspired/delivered ratios of sevoflurane during low-flow anesthesia. Moreover, only Primus^®^ has a decoupling system to prevent barotrauma, which is caused by unexpected increases in tidal volume [12]. In the decoupling system, fresh gas is diverted into the RB during inspiration, and then from the RB into the circular system during expiration [7, 12]. This causes a delay in the time to target sevoflurane concentration [7, 12]. Primus^®^ also has a classical piston ventilator, while Perseus^®^ and Zeus^®^ have the blower-driven ventilators with rapid mixing [13, 14].

In the Primus^®^ and Zeus^®^-F, the times to target sevoflurane concentration were longer than needed with Perseus^®^, because (1) the fresh gas inlets were not close to the patient in either Primus^®^ or Zeus^®^, (2) Primus^®^ has a large internal volume, (3) Primus^®^ has a decoupling system, and (4) Zeus^®^ has two modes of vaporization with the direct injection of volatile anesthetics (DIVA; Table 1) [5, 6, 12–14, 17]. The Primus^®^ and Perseus^®^ use a semi-closed circuit system and a bypass vaporizer (out-of-circle vaporization), whereas Zeus^®^ is the multifunctional system that can operate a semi-closed circuit system (out-of-circle vaporization) or closed-circuit system (in-circle vaporization) using the DIVA vaporizer. In Zeus^®^-F mode (out-of-circle vaporization), the injected sevoflurane is mixed with FGF in a mixing chamber next to the ventilator; conversely, in Zeus^®^-A (in-circle vaporization), the injected sevoflurane is vaporized immediately into the internal circuit, independent of the FGF [5, 6, 14]. Zeus^®^-A allows TCA, with rapid control of sevoflurane concentration by blower-driven ventilation and DIVA, whereas Zeus®-F introduces a delay to the mixing process in the chamber next to the DIVA vaporizer [5, 6, 15].

Before the investigation, we suspected that Zeus^®^-A would require a shorter time to target sevoflurane concentration than Zeus^®^-F; however, at an FGF 3 L/min, the opposite was true. One previous study showed that Zeus^®^-A routinely nears the target setting of sevoflurane early [5]. Thus, to avoid exceeding the target sevoflurane concentration, the AM lowers the speed of the blower-driven ventilator to gradually reach the target concentration [5]. In the present study, Zeus^®^-A reached a sevoflurane concentration of approximately 3.6% very rapidly; there was then a considerable delay before the target sevoflurane concentration of 4% was reached (Fig. 1). With respect to the time to target sevoflurane concentration in Zeus^®^-F and Zeus^®^-A, the blower-driven ventilator with DIVA had a greater effect on the time at FGF 0.5 and 1 L/min, but the FGF rate itself had a greater effect on the time at an FGF 3 L/min,

The Perseus^®^, the latest AM from Dräger, has a blower-driven ventilator and a small internal volume; the fresh gas inlet is close to the patient and it has a classic out-of-circle vaporizer. However, it does not have a decoupling system or a DIVA vaporizer [17]. In clinical practice, rapid rises in target inhalational concentration can be achieved using volatile induction and maintenance anesthesia (VIMA). In such cases, it is better to use a high FGF rate and a high vaporizer setting, as well as an AM with a small internal volume, a blower-driven ventilator, a fresh gas inlet that is close to the patient, and no decoupling system [18].

One limitation of this study was that it did not involve anesthetic uptake by humans. Furthermore, because we measured the sevoflurane concentration at the Y-piece, the recirculated circuit gas may not have mixed adequately. Nevertheless, the results from our study using a test lung will be valuable and helpful in understanding the differences among the various AMs, as well as in developing a new AM in the future.

## Conclusions

In conclusion, the time taken to reach the target concentration was affected by the FGF rate, as well as by the composition and configuration of the AM. Apart from the main factors, such as the rate of FGF rate and the internal volume of the AM, the time to target sevoflurane concentration was also affected by other equipment-factors in the AM: differences in the proximity of the fresh gas inlet to the patient, the presence or absence of a decoupling system, and the use of a blower-driven ventilator with a DIVA vaporizer.

## Abbreviations

AM: Anesthesia machine
DIVA: Direct injection of volatile anesthetic
FGF: Fresh gas flow
MAC: Minimum alveolar concentration
RB: Reservoir bag
TCA: Target-controlled anesthesia
Zeus^®^-A: Zeus^®^ auto-mode
Zeus^®^-F: Zeus^®^ fresh gas mode
